# Inhibitory Effect and Mechanism upon Glucose-Insulin-Potassium Administration on Postpartum Mice with Uterine Cramping Pain

**DOI:** 10.1007/s43032-024-01579-8

**Published:** 2024-05-14

**Authors:** Guiying Yang, Zonghong Long, Fang Chen, Xiaohang Bao, Dukun Zuo, Hong Tang, Zhuoxi Wu, Hong Li

**Affiliations:** Department of Anesthesiology, Second Affiliated Hospital of Army Medical University, No.183 Xinqiao Street, 400037 Shapingba, Chongqing, China

**Keywords:** Uterine cramping pain, Inadequate analgesia, GIK, Postpartum mice

## Abstract

This study aimed to explore the effect of glucose-insulin-potassium (GIK) on postpartum uterine cramping pain(UCP) in mice and the possible underlying mechanisms. Thirty full-term pregnancy C57BL/6 mice, within 6 h after spontaneous labor, the mice were randomly assigned into the following three groups: the control group (group C), the oxytocin group (group O), and the GIK plus oxytocin group (group G). Group G and group O were administered GIK and normal saline, respectively, and 10 min later, oxytocin was injected intraperitoneally; group C received normal saline twice. The pain scores of the mice were assessed after establishment of the postpartum UCP model. The differential expressions of energy metabolism and oxidized lipid metabolites in the uterus were analyzed. The behavioral scores in group G were significantly lower than those in group O (*P* < 0.05).When compared to group O, group G showed a significant increase in ATP levels (*P* = 0.046), and group G exhibited elevated levels of amino acids, including L-glutamine, L-aspartic acid, and ornithine. Additionally, phosphate compounds (2-phosphoglyceric acid and 3-phosphoglyceric acid) showed elevated levels. When compared to group O, group G exhibited a decrease in 19R-hydroxy PGF_2α_, an increase in 9,10-EpOME and 12,13-EpOME, and a decrease in trans-EKODE-E-Ib. Additionally, group G showed an elevation in 16,17-EpDPE and 8-HDoHE. This study confirms the analgesic effect of GIK during postpartum oxytocin infusion. Metabolomics and glycolysis product analysis suggest that GIK’s alleviation of UCP is associated with its enhancement of glycolysis and the influence of phenylalanine synthesis, aspartate metabolism, and arginine synthesis pathways. Additionally, the effects of GIK appears to be linked to its influence on the linoleic acid metabolic pathway.

**Supplementary Information:**

The online version contains supplementary material available at 10.1007/s43032-024-01579-8.

## Introduction

Uterine cramping pain (UCP) is a significant contributing factor to inadequate analgesia following a cesarean delivery [1]. Following vaginal delivery, UCP incidence remains high, with up to 47% of puerperas reporting significant UCP within 48 h post-delivery, especially in multiparous women [2].Currently, oxytocin is routinely used in clinical practice to strengthen uterine contraction and prevent postpartum bleeding, which exacerbates UCP. Non-steroidal drugs have a relieving effect on UCP, but owing to concerns about their safety during breastfeeding, they are not widely used, especially in China [3]. Meanwhile, some studies have found that tenoxicam has no obvious analgesic effect on UCP in women who underwent repeated cesarean sections (NRS score decreased by 1.1) [4]. Therefore, it is necessary to determine new methods for treating UCP to achieve the best analgesic effect with the lowest side effects.

Previous studies have revealed that uterine ischemia/hypoxia is one of the mechanisms underlying UCP [5–6]. Intermittent contractions of the uterine smooth muscles compress the blood vessels that cross the uterine muscle wall, leading to local tissue ischemia and hypoxia. A mouse studyfurther corroborated this by showing that an increase in the uterine pressure, caused by oxytocin, decreased uterine oxygenation by 38%, even in the absence of inflammatory factors. Additionally, inducing uterine contractions using platelet-activating factor decreases uterine perfusion by 40 ± 8%, resulting in extremely low oxygen partial pressure (9.4 ± 3.4 mm Hg PaO2), thereby causing UCP [7].

Glucose-insulin-potassium (GIK) is a medication known for its ability to enhance tissue energy metabolism and provide ischemia/hypoxia protection. It has been used in clinical practice since the 1970s and is commonly utilized for myocardial protection and the treatment of angina pectoris. Multiple studies have indicated its significant efficacy [8–11]. Its mechanism of action involves improving myocardial glucose metabolism, promoting glycolysis, increasing high-energy phosphate levels, and effectively clearing pain-inducing substances such as lactate, the end product of glycolysis. This process reduces intracellular lactate and H^+^ accumulation, leading to the improvement of angina pectoris symptoms. GIK has also been reported to provide protection against ischemic injuries in the skeletal muscles, brain, and intestines [12–14] and can alleviate pain following skeletal-muscle ischemic injury [14].

Given the promising indications of GIK in improving energy metabolism in other organ tissues and providing ischemic protection, we hypothesized that GIK may enhance uterine energy metabolism, increase uterine tolerance to ischemia/hypoxia, reduce the accumulation of pain-inducing substances, and consequently ameliorate UCP. We had observed the effect of GIK in relieving UCP after cesarean section in our previous clinical studies [15]. However, the mechanism remains to be further studied.

To explore the role and mechanism of GIK in relieving UCP, in this study, we used oxytocin to establish a UCP model in postpartum mice and performed metabolomics analyses to identify the differential metabolites in the uterus of these mice, after receiving oxytocin or oxytocin plus GIK. This study focused on targeted metabolomics studies of energy metabolism and oxidized lipid metabolism. The reason is that GIK is a medication known to improve tissue energy metabolism, which can enhance the energy metabolism of tissues such as skeletal muscle, brain, and intestines during ischemic conditions [12–14]. Simultaneously, substances associated with UCP are primarily arachidonic acids, such as PGE_2_ and PGF_2а_. Therefore, the potential of GIK to alleviate UCP may be associated with alterations in energy metabolism and oxidative lipid metabolism products.

## Materials and Methods

This study was approved by the Animal Welfare Ethics Review Committee of the Third Military Medical University and met the animal welfare requirements.

(ID: AMUWEC20200266).

### Model Building and Grouping

Thirty full-term pregnant C57BL/6 mice were obtained from the Animal Experimental Center of the Army Medical University. Within 6 h after natural delivery, the mice were randomly assigned into the following three groups of 10 mice each: the control group (group C), the oxytocin group (group O), and the GIK plus oxytocin group (group G). The weights of the mice were measured before the experiment. Group G was intraperitoneally administered GIK (10% sugar water 250 mL + insulin 8 IU + potassium 0.8 g) at a dose of 57.6 mL/kg, based on previous studies that reported a dose of 40 mL/kg in rats [16], and a mouse dose of 1.44 times higher than that in rats [17]. Groups C and O were administered an equal volume of normal saline. Ten minutes after the injection, oxytocin (100 IU/kg) was administered intraperitoneally to group G and group O, while group C received an equal volume of normal saline. Pain behavior was observed in the mice for 1 h after inducing the postpartum UCP model. eFig.1 in Supplement shows the construction of the UCP model and the overall experimental process.

### Observing Pain Behavior

Normal behavior was scored as 0. Less symptomatic painful behaviors such as isolation/immobility and abdominal wall licking were scored as 1. More severe painful behaviors, including extreme trunk and limb extension and unusual hunched posture, were scored as 2 [7]. Behavioral changes within 1 h after intraperitoneal injection of oxytocin were recorded once every 10 min, and the most severe pain behavior in each period was recorded as the final pain behavior. The pain scorer was blinded to the grouping of the mice.

### Uterine Sample Acquisition

Following the observation of pain behavior, six of mice in each group were anesthetized until the normal reflex disappeared. And then uteri were immediately removed by a laparotomy, and finally mice were sacrificed by cervical dislocation. The specimens were stored at − 80 °C. Liquid chromatography tandem mass spectrometry (LC-MS [SCIEX Corporation, USA]) was used for the accurate and quantitative detection of different metabolites involved in energy metabolism and oxidized lipid metabolism.

### Paraffin Section Preparation and HE Staining

Following the pain behavior observation, three of mice in each group were anesthetized until the normal reflex disappeared. And then the mice were perfused, and their uteri were fixed with formalin to prepare for histopathological analyses, and finally mice were sacrificed by cervical dislocation. The uterine tissue was paraffin-embedded and stained with hematoxylin eosin (HE, Beijing Soleibao Technology Co., LTD., China)for further examination. The resulting samples were analyzed and imaged using an Olympus CX31 microscope (Olympus Corporation, Japan).

### Metabolomics Targeting for Metabolite Detection and Screening

(See Appendix 1 in Supplement)

### Detection of glycolysis-Related Substances

(See Appendix 2 in Supplement)

### Statistical Analysis

All data were analyzed by SPSS 26.0. Statistical significance was determined by a bilateral *P <* 0.05. Continuous variables with normal distribution were presented as mean ± standard deviation, while those with non-normal distribution were expressed as median and interquartile ranges (IQRs).

Normally distributed continuous data of three groups were compared using analysis of variance (ANOVA), and Tukey’s post hoc analysis was performed for pairwise comparisons between continuous variables of each pair of groups. The onset time and duration of abdominal pain were analyzed using t-tests (group C showed no behavioral changes in pain and had no onset time and duration). A generalized linear model was used for the overall comparison of non-normally distributed continuous variables (pain behavioral scores) among the three groups. The Mann–Whitney U test was used for pain behavioral scores and comparisons at different time periods, and *P* < 0.01667 was used to indicate statistically significant differences.

## Results

### Behavioral Results

As shown in Table 1, the analysis conducted using the generalized linear model revealed significant effects related to the group, time, and an interaction between time and group (all, *P* < 0.001) on the behavioral scores of the mice. During the 30–40-minute, 40–50-minute, and 50–60-minute time intervals, the behavioral scores in group G were significantly lower than those in group O (0.0 [IQR, 0.0–1.0] vs. 2.0 [IQR, 1.8-2.0], *P* = 0.002; 0.0 [IQR, 0.0–1.0] vs. 1.0 [IQR, 1.0–2.0], *P* = 0.001; and 0.0 [IQR, 0.0–1.0] vs. 1.0 [IQR, 1.0–2.0], *P* = 0.005, respectively). Furthermore, the duration of pain in group G was significantly shorter than that in group O (39.5 ± 18.8 min vs. 56.1 ± 7.6 min, *P* = 0.018), and there was no significant difference in pain onset time between the two groups (105.4 ± 41.3 s vs. 79.6 ± 33.3 s, *P* = 0.147).

**Table 1:** Sum of pain behavior scores in different time periods

	Group C (*n* = 10)	Group O (*n* = 10)	Group G (*n* = 10)	*P* values between three groups	*P* values compared between groups		
					O vs.G	C vs.O	C vs.G
0–10 min	0.0 (0.0,0.0)	1.5(1.0,2.0)	1.5(1.0,2.0)	< 0.001	0.831	< 0.001	< 0.001
10–20 min	0.0 (0.0,0.0)	2.0(1.0,2.0)	1.0(0.8,1.3)	< 0.001	0.836	< 0.001	0.001
20–30 min	0.0 (0.0,0.0)	2.0(1.0,2.0)	1.0(0.0,1.0)	< 0.001	0.011	< 0.001	0.005
30–40 min	0.0 (0.0,0.0)	2.0(1.8,2.0)	0.0(0.0,1.0)	< 0.001	0.002	< 0.001	0.068
40–50 min	0.0 (0.0,0.0)	1.0(1.0,2.0)	0.0(0.0,1.0)	< 0.001	0.001	< 0.001	0.146
50–60 min	0.0 (0.0,0.0)	1.0(1.0,2.0)	0.0(0.0,1.0)	< 0.001	0.005	0.001	0.146

Data were presented median (interquartile range)

### Morphological Features Results

**Fig. 1:**
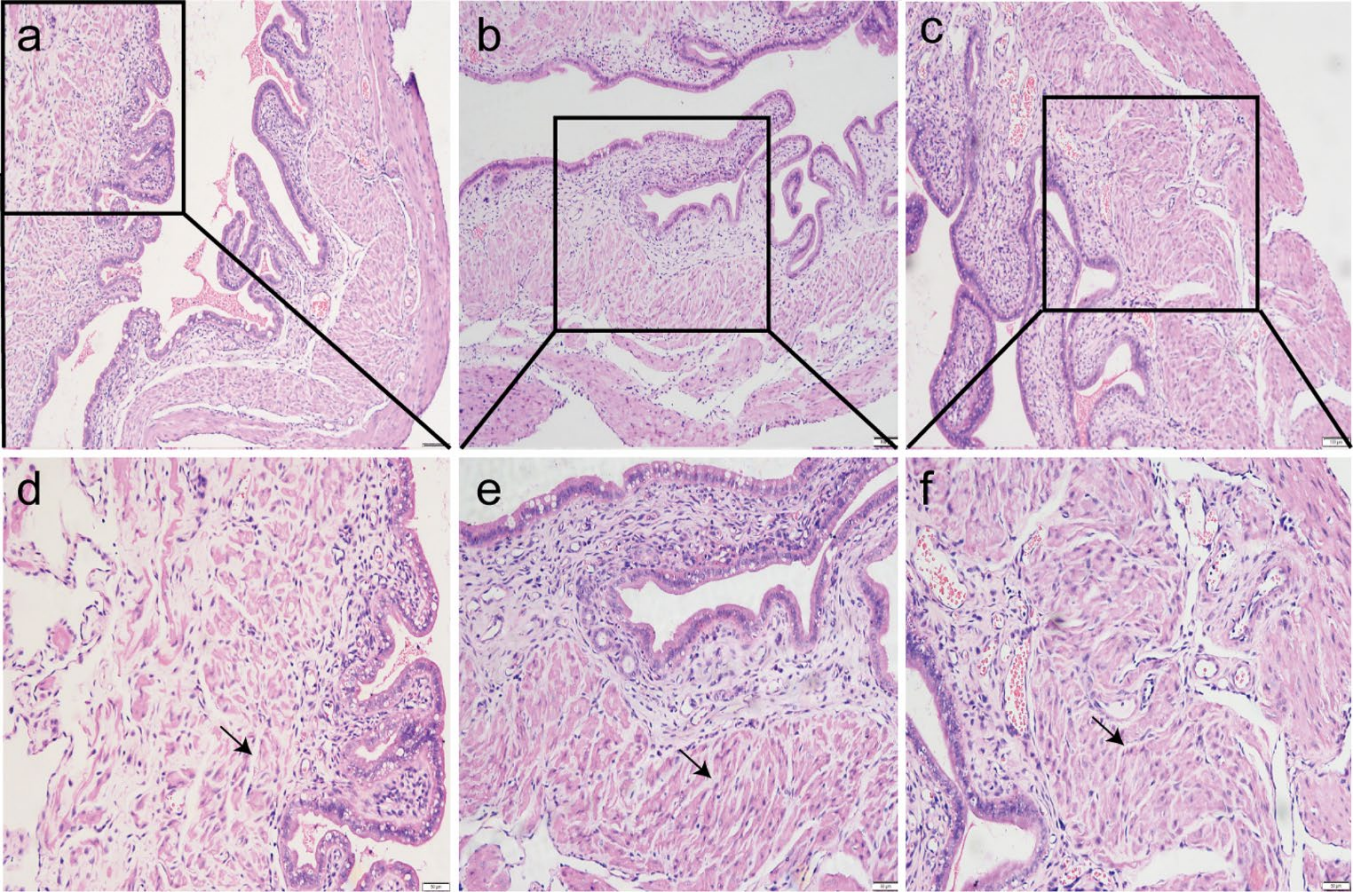
Comparisons of morphological features of uterine wall muscle (*n* = 3). Group C (**a**); Group O (**b**); Group G (**c**), (HE×100, bar = 100 μm). Group C (**d**); Group O (**e**); Group G (**f**), (HE×200,bar = 50 μm). Arrow indicates the location of the muscle interspace. A separation of uterine wall muscle fibers, widening of the muscle interspace, dilation of blood sinuses, and a state of blood stasis were observed in group C. The muscle fibers were more complete and uniform in group G, with lesser edema in the muscle interspace compared to those in group O. HE, Hematoxylin Eosin

Microscopic analysis of HE-stained uteri showed separation of uterine wall muscle fibers, widening of the muscle interspace, dilation of blood sinuses, and a state of blood stasis in the group C, and no obvious separation of muscle fibers was observed in mice in group G and group O. Muscle fibers in group G were more complete and uniform and had lesser edema in the muscle interspace than those in group O, as shown in Fig. 1.

### Energy Metabolomics Analysis Results

#### Sample Principal Component Analysis(PCA) and Cluster Analysis

**Fig. 2:**
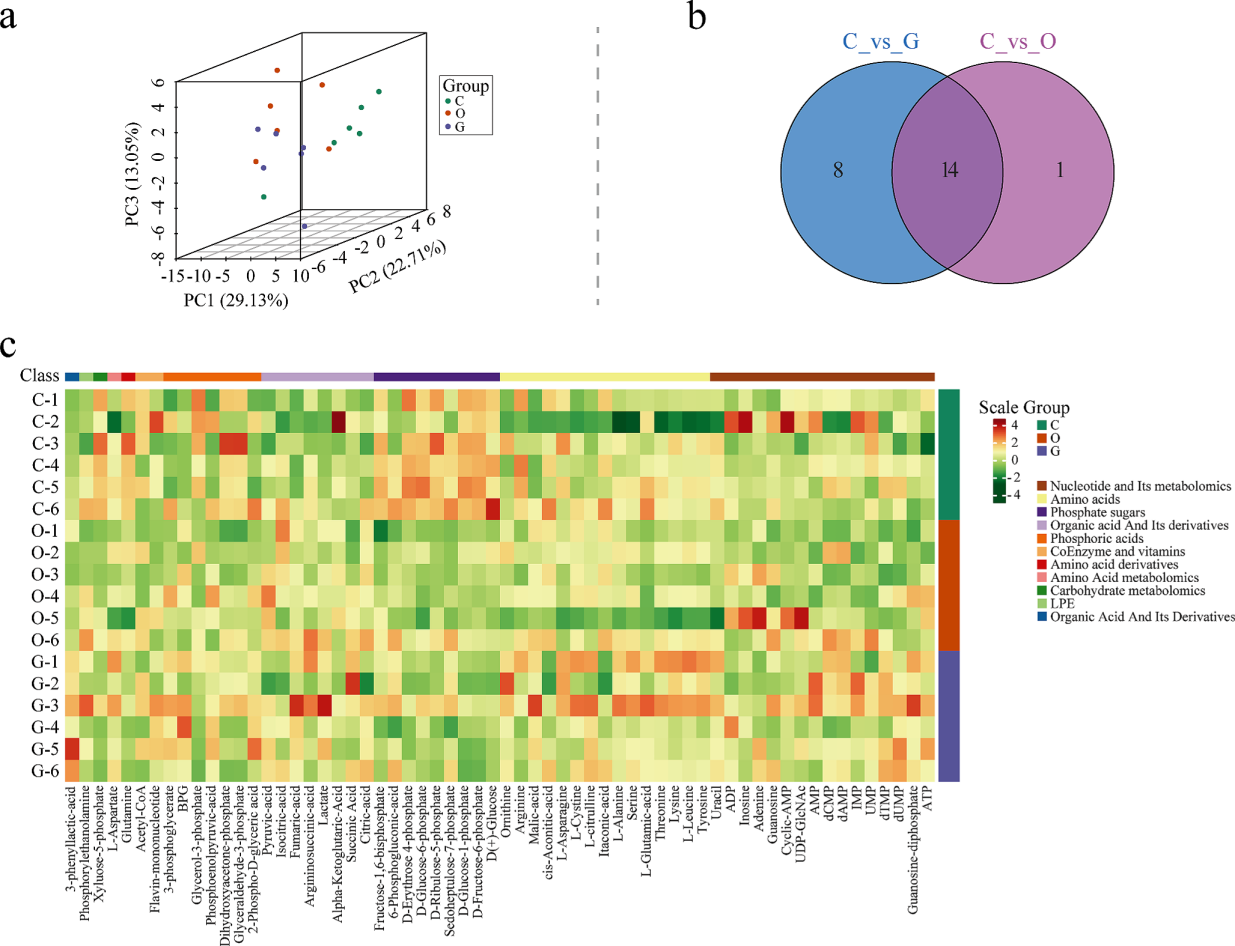
PCA and cluster analysis(*n* = 6).(**a**) PCA scores of mass spectrum data of each group; (**b**) Venn diagram of differential metabolites; (**c**) Heat maps of three groups of energy metabolites. PCA, Sample Principal Component Analysis

PCA of the samples was performed. Results indicated that metabolites in groups C, G, and O showed a separation trend, indicating differences in energy metabolites among the groups, as shown in Fig. 2a. There were 22 distinct metabolites between groups C and group G, 15 unique metabolites between group C and group O, and 14 distinct metabolites between the two comparison groups, as depicted in Fig. 2b. Moreover, cluster analysis was conducted on all samples; Fig. 2c indicates the heat maps of energy metabolites in the three groups. Different metabolites between the groups are shown in Table 2 and eFig.3–5 in supplement. Ten metabolites were increased and 12 metabolites were decreased in group G compared to that in group C. In addition, 12 metabolites were decreased and 3 metabolites were increased in group O compared to that in group C. Furthermore, six metabolites were increased in group G compared to that in group O.

**Table 2:** Different energy metabolites between groups(*n* = 6)

	Compounds	Class	*P*-value	FC	Type
Group C and group G	Glutamine	Amino acid derivative	0.020	0.744	down
	Serine	Amino acid	0.078	1.208	up
	L-citrulline	Amino acid	0.012	1.575	up
	L-Cystine	Amino acid	0.076	1.377	up
	Malic-acid	Amino acid	0.005	1.207	up
	Ornithine	Amino acid	0.030	1.438	up
	Xyluose-5-phosphate	Carbohydrate metabolomics	< 0.001	0.741	down
	3-phenyllactic-acid	Organic acid and its derivatives	0.002	2.139	up
	Lactate	Organic acid and its derivatives	0.014	1.611	up
	Fumaric-acid	Organic acid and its derivatives	0.005	1.389	up
	D-Glucose	Phosphate sugar	0.005	0.425	down
	D-Fructose-6-phosphate	Phosphate sugar	< 0.001	0.641	down
	D-Glucose-1-phosphate	Phosphate sugar	< 0.001	0.649	down
	D-Ribulose-5-phosphate	Phosphate sugar	0.002	0.741	down
	D-Glucose-6-phosphate	Phosphate sugar	0.003	0.811	down
	D-Erythrose4-phosphate	Phosphate sugar	0.004	0.679	down
	Fructose-1,6-bisphosphate	Phosphate sugar	0.013	0.753	down
	Glyceraldehyde-3-phosphate	Phosphoric acids	0.082	0.805	down
	Dihydroxyacetone-phosphate	Phosphoric acids	0.073	0.804	down
	Glycerol-3-phosphate	Phosphoric acids	0.010	0.700	down
	BPG	Phosphoric acids	0.017	7.240	up
	3-phosphoglycerate	Phosphoric acids	0.086	1.436	up
Group C and group O	Glutamine	Amino acid derivative	0.091	0.807	down
	Xyluose-5-phosphate	Carbohydrate metabolomics	3.385	0.758	down
	Lactate	Organic acid and its derivatives	0.008	1.501	up
	Fumaric-acid	Organic acid and its derivatives	0.006	1.238	up
	Pyruvic-acid	Organic acid and its derivatives	0.050	1.281	up
	D-Glucose	Amino acid	0.012	0.526	down
	D-Fructose-6-phosphate	Amino acid	4.345	0.725	down
	D-Glucose-1-phosphate	Amino acid	1.246	0.736	down
	D-Ribulose-5-phosphate	Amino acid	0.003	0.756	down
	D-Glucose-6-phosphate	Amino acid	8.175	0.750	down
	D-Erythrose4-phosphate	Amino acid	0.009	0.730	down
	Fructose-1,6-bisphosphate	Amino acid	0.012	0.673	down
	Glyceraldehyde-3-phosphate	Carbohydrate metabolomics	0.029	0.753	down
	Dihydroxyacetone-phosphate	Carbohydrate metabolomics	0.029	0.753	down
	Glycerol-3-phosphate	Carbohydrate metabolomics	0.066	0.791	down
Group O and group G	L-citrulline	Amino acid	0.052	1.339	up
	L-Asparagine	Amino acid	0.047	1.631	up
	Ornithine	Amino acid	0.064	1.273	up
	3-phenyllactic-acid	Organic acid and its derivatives	0.003	1.936	up
	2-Phospho-D-glyceric acid	Phosphoric acids	0.067	1.507	up
	3-phosphoglycerate	Phosphoric acids	0.054	1.283	up

*Abbreviations* FC, fold change

Screening criteria entailed a *P*-value < 0.1 and the| FC| ≥ 1.2

#### Metabolic Pathway Analysis

**Fig. 3:**
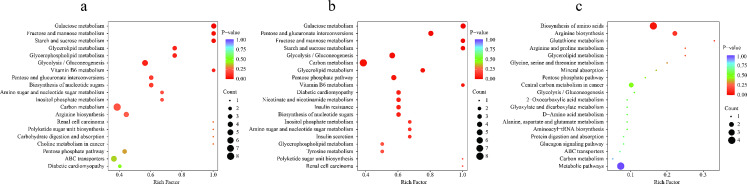
The KEGG enrichment bubble plots for metabolites. The x-axis represents the rich factor for each pathway; the y-axis shows the pathway name; the color of the dots represents the *P*-value; the size of the dots indicates the number of enriched differential metabolites. (**a**) between group G and group C; (**b**) between group O and group C; (**c**) between group G and group O. KEGG, Kyoto Encyclopedia of Genes and Genomes

**Fig. 4:**
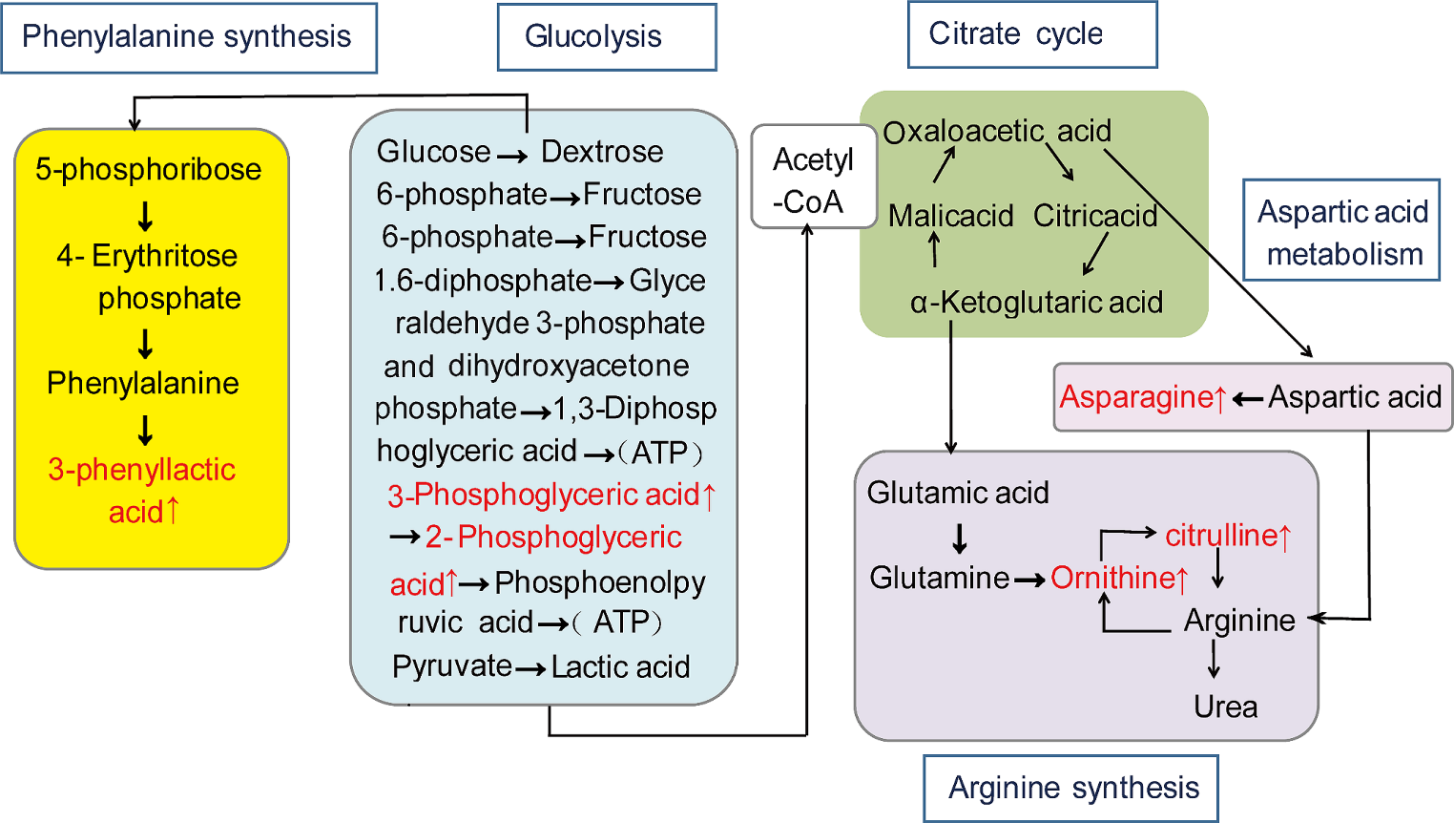
Joint analysis of energy metabolism biomarkers based on enrichment pathway and differential metabolites between group G and group O. The blue box represents metabolite pathways; the red font represents an increase; the green font represents a decrease

As shown in Fig. 3, using the Kyoto Encyclopedia of Genes and Genomes (KEGG) metabolic pathways analysis, seven pathways were identified in group C and group G (*P* < 0.05), including galactose metabolism, fructose and mannose metabolism, starch and sucrose metabolism, glycerolipid metabolism, glycerophospholipid metabolism, glycolysis/gluconeogenesis, and vitamin B6 metabolism. Between group C and group O, nine pathways were identified (*P* < 0.05), including galactose metabolism, pentose and glucuronate interconversions, fructose and mannose metabolism, starch and sucrose metabolism, glycolysis/gluconeogenesis, carbon metabolism, glycerolipid metabolism, pentose phosphate pathway, and vitamin B6 metabolism. Only one pathway was identified between group O and group G (*P* < 0.05): biosynthesis of amino acids. Additionally, glycolysis/gluconeogenesis and arginine biosynthesis aspartic acid and glutamic acid metabolism pathways were enriched. Figure 4 illustrates the association between differential energy metabolites and potential biomarkers, enriched in pathways between group G and group O. Significant changes in metabolites were associated with glycolysis, phenylalanine synthesis, aspartic acid metabolism, and arginine synthesis.

### Results of Oxidative Lipid Metabolomics Analysis

#### Sample PCA and Cluster Analysis

**Fig. 5:**
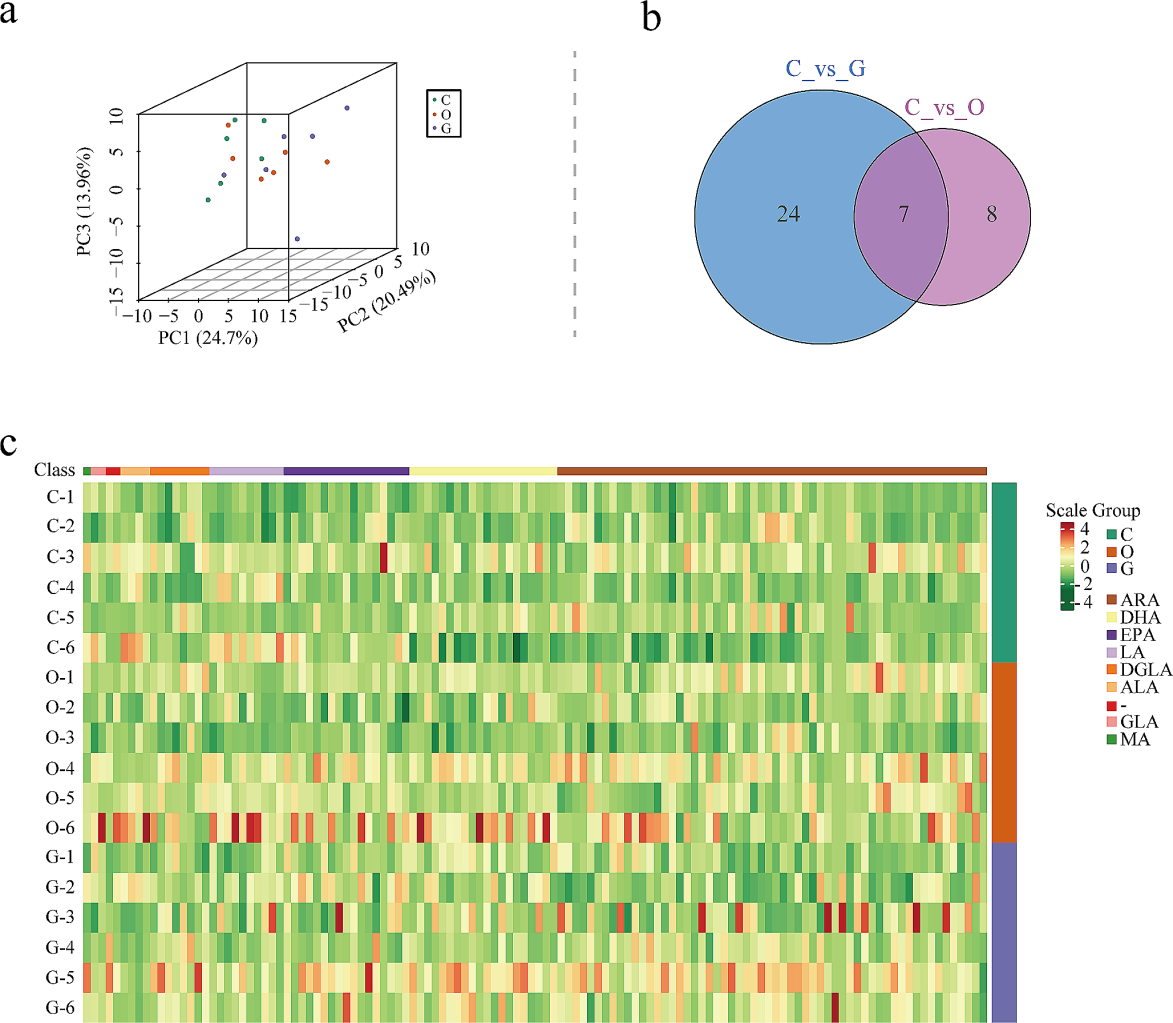
PCA and cluster analysis(*n* = 6). (**a**) PCA scores of mass spectrum data of each group; (**b**) Venn diagram of differential metabolites; (**c**) Heat maps of oxidized lipid metabolites in three groups. PCA, principal component analysis

PCA was performed on the samples. Results showed a separation trend of metabolites among groups C, G, and O, indicating differences in oxidized lipid metabolites among the groups, as shown in Fig. 5a. There were 31 different metabolites between group C and group G and 15 different metabolites between group C and group O; seven different metabolites were in common between the two comparison groups, as shown in Fig. 5b. Cluster analysis was performed on all the samples. Figure 5c shows the heat maps of oxidized lipid metabolites in the three groups. Different metabolites between the groups are shown in Table 3 and eFig.7–9 in supplement. Group G showed a significant increase in 25 metabolites and a decrease in 6 metabolites compared to group C. Group O showed an increase in 15 metabolites compared to group C. Moreover, group G showed an increase in 4 metabolites and a decrease in 2 metabolites compared to group O.

**Table 3:** Different oxidized lipid metabolites between groups(*n* = 6)

	Compounds	Class	*P*-value	FC	Type
Group C and group G	9-OxoOTrE	ALA	0.080	0.402	down
	20-HETE	ARA	0.056	0.710	down
	11,12-DiHET	ARA	0.025	1.256	up
	LXB_4_	ARA	0.008	1.474	up
	20-COOH-LTB_4_	ARA	0.093	1.216	up
	19R-hydroxyPGF_2α_	ARA	0.011	0.705	down
	5,6-DiHETrE	ARA	0.025	1.786	up
	5-isoPGF_2VI_	ARA	0.098	1.681	up
	12-epi LTB_4_	ARA	0.079	1.436	up
	8,9-EET	ARA	0.081	2.129	up
	5,6-EET	ARA	0.092	0.771	down
	12-HETE	ARA	0.006	1.490	up
	16-HETE	ARA	0.035	1.230	up
	14,15-EET	ARA	0.027	1.322	up
	tetranor-12 S-HETE	ARA	0.091	4.345	up
	11,12-EET	ARA	0.092	1.380	up
	8 S-HETrE	DGLA	0.014	1.460	up
	TXB_1_	DGLA	0.076	1.556	up
	16,17-EpDPE	DHA	0.016	1.436	up
	10-HDoHE	DHA	0.008	1.659	up
	19,20-DiHDPE	DHA	0.053	0.771	down
	8-HDoHE	DHA	0.028	1.344	up
	4-HDoHE	DHA	0.005	1.548	up
	19,20-EpDPE	DHA	0.021	1.488	up
	20-HDoHE	DHA	0.094	1.287	up
	17,18-EpETE	EPA	0.069	1.231	up
	9-HEPE	EPA	0.006	2.062	up
	EPA	EPA	0.049	1.219	up
	12-HEPE	EPA	0.007	2.121	up
	12,13-EpOME	LA	0.057	1.309	up
	9,10-DiHOME	LA	0.095	0.447	down
Group C and group O	5 S,15 S-DiHETE	ARA	0.084	2.054	up
	LXB_4_	ARA	0.056	1.541	up
	6-trans-LTB_4_	ARA	0.078	2.366	up
	8-iso-PGF_2α_	ARA	0.089	1.306	up
	15-keto-PGF_2α_	ARA	0.055	1.915	up
	8,9-EET	ARA	0.067	1.558	up
	12-HETE	ARA	0.017	1.425	up
	15-HETE	ARA	0.028	1.427	up
	tetranor-12 S-HETE	ARA	0.031	3.649	up
	5-HETE	ARA	0.062	1.244	up
	8 S-HETrE	DGLA	0.051	1.410	up
	10-HDoHE	DHA	0.029	1.650	up
	4-HDoHE	DHA	0.011	1.432	up
	11,12-DiHETE	EPA	0.072	1.920	up
	18-HEPE	EPA	0.026	1.712	up
Group O and group G	19R-hydroxy PGF_2α_	ARA	0.059	0.600	down
	16,17-EpDPE	DHA	0.074	1.288	up
	8-HDoHE	DHA	0.058	1.295	up
	9,10-EpOME	LA	0.094	1.288	up
	12,13-EpOME	LA	0.047	1.227	up
	trans-EKODE-E-Ib	LA	0.093	0.688	down

*Abbreviations* FC, fold change; ALA, linolenic acid; ARA, Arachidonic acid; DGLA, Dihomog-linolenic acid; LA, Linoleic acid (LA); EPA, Eicosapentaenoic acid; DHA, Docosahexaenoic acid

Screening criteria entailed a *P*-value < 0.1 and the| FC| ≥ 1.2

### Metabolic Pathway Analysis

**Fig. 6:**
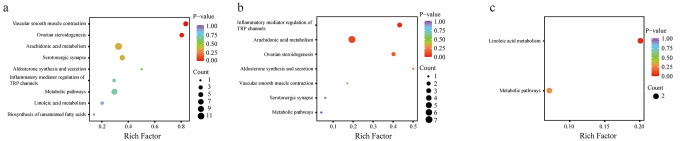
KEGG enrichment bubble plots for metabolites. The x-axis represents the Rich Factor for each pathway; the y-axis shows the pathway name; the color of the dots represents the *P*-value; the size of the dots indicates the number of enriched differential metabolites. (**a**) between group G and group C; (**b**) between group O and group C; (**c**) between group G and group O. KEGG, Kyoto Encyclopedia of Genes and Genomes

**Fig. 7:**
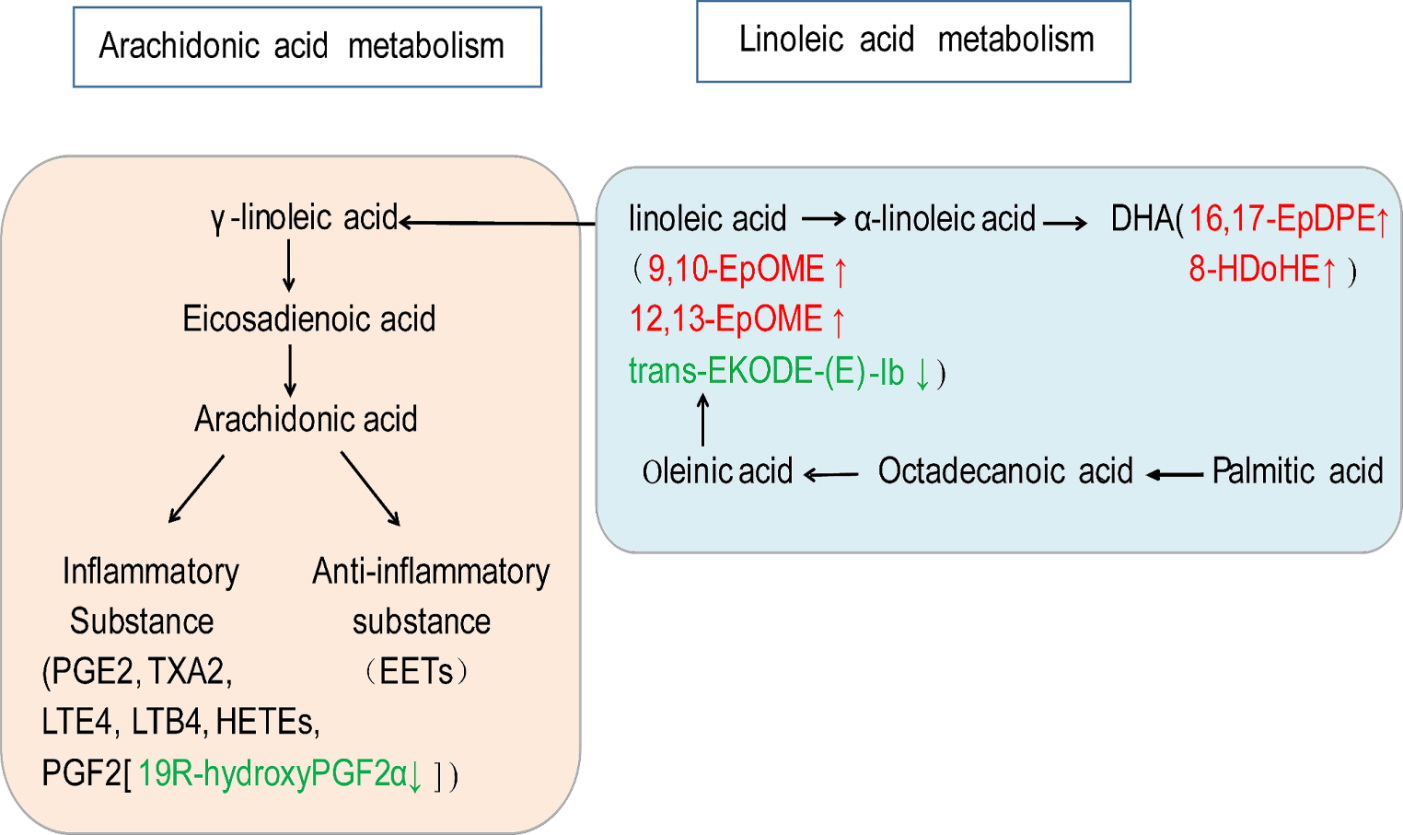
Joint analysis of energy metabolism biomarkers based on enrichment pathway, and differential metabolites between group G and group O. The blue box represents metabolite pathways; the red font represents an increase; the green font represents a decrease

Using KEGG metabolic pathways analysis, two pathways were identified with *P* < 0.05 between group C and group G, namely vascular smooth muscle contraction and ovarian steroid generation. Similarly, two pathways were found with *P* < 0.05 between group C and group O, namely the inflammatory mediator regulation of TRP channels and arachidonic acid metabolism. Furthermore, one pathway with *P* < 0.05 was identified between group O and group G, which was linoleic acid metabolism. Additionally, the metabolic pathway was enriched, as depicted in Fig. 6. Figure 7 illustrates the correlation between the differential oxidative lipid metabolites and the enrichment pathway potential biomarkers between group G and group O. Notably, metabolites with significant changes were related to the metabolism of linoleic acid metabolism.

### Detection of Glycolysis-Related Substances

**Fig. 8:**
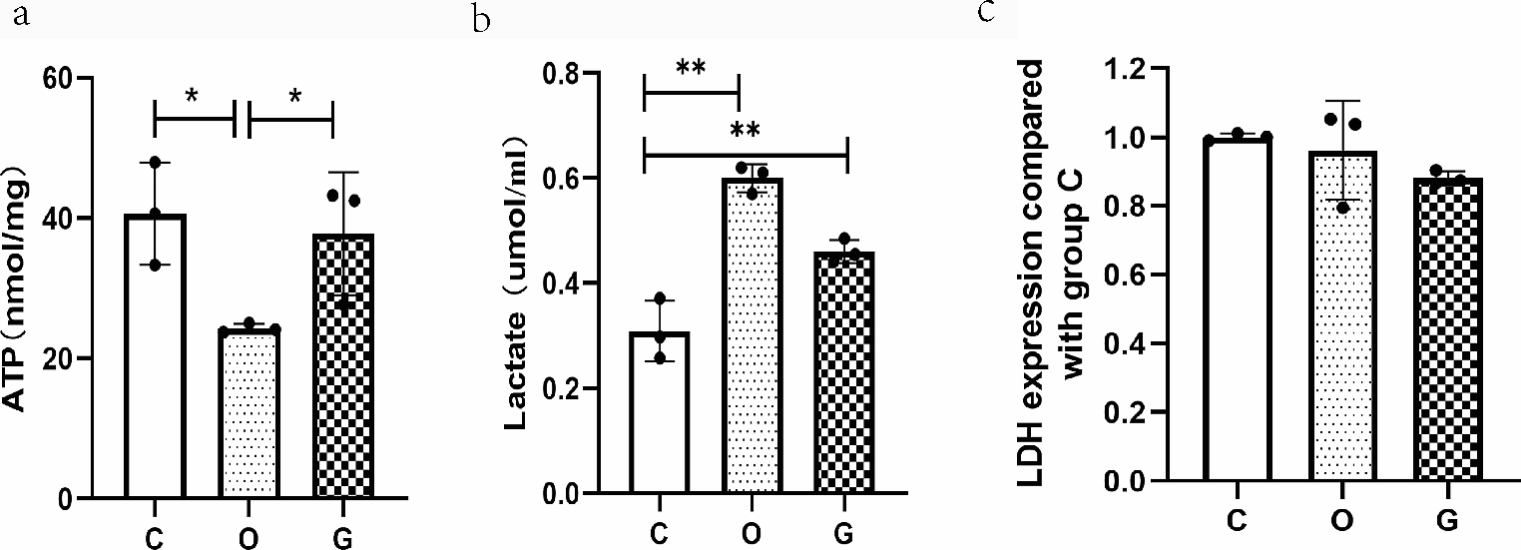
(**a**) ATP level; (**b**) Lactate concentration; (**c**) LDH content relative to group C (*n* = 3). *P < 0.05, **P < 0.01. ATP, Adenosine triphosphate; LDH, Lactate dehydrogenase

Compared to that in group C, the ATP levels in uterine tissues of group O decreased (24.30 ± 0.66 nmol/mg vs. 40.62 ± 7.29 nmol/mg, *P* = 0.023). Compared to group O, group G showed a significant increase in ATP levels (37.78 ± 8.75 nmol/mg vs. 24.30 ± 0.66 nmol/mg, *P* = 0.046). Both group G and group O exhibited elevated lactate levels compared to group C (0.60 ± 0.07 µmol/mg vs. 0.12 ± 0.04 µmol/mg, *P* = 0.023 and 0.87 ± 0.32 µmol/mg vs. 0.12 ± 0.04 µmol/mg, *P* = 0.003, respectively). There was no statistically significant difference in LDH content among the three groups (*P* > 0.05), as shown in Fig. 8.

## Discussion

The results of the pain behavior analysis conducted in our study indicated that GIK decreased the pain behavior score, suggesting that GIK alleviated UCP in mice. Additionally, the results of the energy metabolism analysis showed that several compounds in the uterine tissue of mice treated with the combination of GIK and oxytocin, including L-citrulline, L-asparagine, ornithine, 3-phenyllactic acid, 2-phospho-D-glyceric acid, and 3-phosphoglyceric acid, increased compared to those in mice treated with oxytocin alone. Furthermore, the oxidative lipid metabolomics results indicated that the combination of GIK and oxytocin treatment decreased the levels of 19R-hydroxy PGF_2α_, while increasing the levels of docosahexaenoic acid (DHA),16,17-EpDPE and 8-HDoHE, and linoleic acid (9,10-EpOME and 12,13-EpOME) in the mouse uterine tissue. Conversely, the levels of linolenic acid (trans-EKODE-E-Ib) decreased in the same group compared to those in the group treated with oxytocin alone. The results of glycolysis product analysis indicated a decrease in ATP levels and an increase in lactate levels within the uterine tissue following oxytocin administration. However, the additional administration of GIK led to a recovery in ATP levels. This suggests that GIK improves uterine energy metabolism without increasing lactate accumulation. Overall, these results suggest that GIK increased the production of amino acids and DHA, while decreasing the production of prostaglandin F2α analogs. Additionally, GIK promoted glycolysis and affected the production of linoleic acid. To the best of our knowledge, this study is the first to investigate the principal metabolite responsible for UCP caused by oxytocin and how GIK relieves UCP from a metabolomics perspective.

Figure 4 illustrates the glycolysis process, which involves several intermediates, including glucose-6-phosphate, fructose-6-phosphate, fructose-1,6-diphosphate, glyceraldehyde 3-phosphate, and dihydroxyacetone phosphate, among others. Compared to oxytocin only, the combination of GIK and oxytocin showed significant elevation in the levels of 2-phospho-D-glyceric acid and 3-phosphoglyceric acid, which are intermediates in glycolysis. Additionally, the results of glycolytic substance analysis indicated that compared to group O, group G exhibited an increase in ATP levels. These findings collectively suggest that, compared to using oxytocin alone, GIK promotes glycolysis and improves uterine energy supply.

Previous studies have reported that GIK has the potential to alleviate angina pectoris [12, 13] and skeletal muscle pain following ischemia [14].GIK provides glucose, and the insulin within it facilitates glucose uptake and utilization by cells, thus supplying energy to ischemic tissues. Research in late-term pregnant rats revealed an increase in glycogen levels in the uterine muscle layer, enhanced glycogen breakdown during labor to provide high energy, increased sensitivity of the muscle layer to insulin, and increased glucose uptake in the presence of insulin [18].This suggests that insulin and glucose play a crucial role in the energy supply to the laboring uterus. Furthermore, Anai et al. reported that pregnant women with LDH deficiency experience reduced ATP generation during uterine smooth muscle glycolysis, leading to increased UCP [19]. Disruptions in glycolytic metabolism can cause UCP and indicate that enhancing glycolysis may improve uterine energy metabolism and alleviate UCP. Based on these clues, the relief of UCP by GIK may be associated with its promotion of glycolysis and improvement of uterine energy metabolism. Morphological results of the uteri also suggest that group G exhibited more intact uterine muscle fibers and reduced interstitial edema, which may also be related to GIK’s improvement of uterine energy metabolism and mitigation of ischemic injury. However, this study did not find any differences in LDH levels among the three groups, indicating that the process by which GIK promotes glycolysis may occur upstream of lactate production. This warrants further research to provide direct evidence.

Our study found that the addition of GIK increased the levels of L-citrulline, L-asparagine, L-aspartic acid, and DL-3-phenyllactic acid in the uterine tissue of mice, compared to those in mice treated with oxytocin alone. These amino acids have been shown to improve tissue microcirculation [20–24].L-citrulline functions similarly to a nitric oxide (NO) supplement and can be converted to L-arginine in the body. L-arginine primarily acts through the arginine-NO pathway and generates citrulline and NO [20] in vivo. The release of NO can cause vasodilation, promote blood circulation, alleviate ischemic hypoxic injury [21], and reduce excessive oxygen consumption in muscles. Taking citrulline supplements can enhance athletic performance, increase exercise tolerance, and even alleviate post-exercise muscle soreness [22].The increase in L-citrulline levels in this study, after GIK administration, suggests that GIK may expand uterine blood vessels through the arginine-NO pathway, thereby increasing the tolerance of the uterus to ischemia and hypoxia. Ornithine can combine with carbamoyl phosphate to produce citrulline, thereby ameliorating uterine ischemia and hypoxia through the same pathway as that of citrulline. L-asparagine has peripheral vascular dilatation effects [23]. Xu et al. found that asparagine supplementation in mice significantly increased intracellular glycolysis intermediates (1,6 diphospho-fructose,6 phospho-fructose) and up-regulated the protein levels of key glycolysis enzymes (hexokinase 2, pyruvate kinase, phosphofructokinase ). They believed that asparagine could activate glycolysis, improving systemic metabolism [24]. Therefore, L-asparagine may improve uterine energy metabolism by dilating uterine blood vessels and promoting uterine glycolysis. Further, 3-phenyllactic acid is a derivative of 3,4-dihydroxyphenyllactic acid, a bioactive compound from the Chinese medicinal herb “Danshen.” It has pharmacological effects similar to those of salvianolic acid and can inhibit platelet aggregation and thrombosis, improve microcirculation, and has therapeutic effects on ischemic heart disease [25]. Therefore, 3-phenyllactic acid may also improve uterine microcirculation. In conclusion, these amino acids may play an important role in improving uterine microcirculation and promoting uterine energy metabolism.

Our analysis also revealed an increase in lactic acid levels, an end product of glycolysis, in both group G and group O compared to that in group C, thereby confirming that pressure on the uterine blood vessels during uterine contractions may lead to an increase in lactate levels due to uterine ischemia/hypoxia. However, there was no significant difference between group G and group C, indicating that although group G showed enhanced glycolysis, there was no significant lactic acid accumulation. Therefore, other metabolic pathways may be involved in lactic acid metabolism. As previously mentioned, compounds such as L-citrulline, ornithine, L-asparagine, and 3-phenyllactic-acid can improve microcirculation, which may accelerate blood flow in the uterus, and facilitate the removal and metabolism of lactic acid. However, further research is needed to clarify the specific mechanisms of this process.

Furthermore, we observed that mice in group O and group G exhibited changes in pain behavior after receiving oxytocin, with higher pain behavior scores than those in mice in group C, indicating successful creation of the UCP model. The mechanism of UCP induced by oxytocin involves the activation of G-protein-coupled oxytocin receptors in the uterus, leading to a significant increase in cellular Ca^2+^ concentration and prostaglandin synthesis. This results in the contraction of uterine smooth muscle, cell membrane damage, and UCP. Results of oxidative lipid metabolism analysis indicated that the expression of 10 arachidonic acids in group O was increased compared to that in group C. Among them, 5 S,15 S-DiHETE was shown to promote the contraction, migration, and proliferation of smooth muscle cells, and plays a crucial role in inducing inflammation and vasoconstriction [26]. Additionally, 6-trans-LTB_4_, 8-iso-PGF_2α_, and 15-keto-PGF_2α_ were identified as powerful vasoconstrictors of contractile smooth muscle and pro-inflammatory substances, leading to intense contraction of uterine smooth muscle, ischemia and hypoxia, ultimately causing UCP. These results establish arachidonic acid to be the primary cause of UCP induced by oxytocin.

Linoleic acid is an essential fatty acid that plays a vital role in synthesizing phospholipids and maintaining the integrity of cell membranes. It also serves as a precursor of leukotrienes and prostaglandins [27]. Figure 7 shows that, compared to group O, group G had increased levels of linoleic acid (9, 10-epome, 12, 13-epome) and decreased levels of linolenic acid (trans-EKODE-E-Ib); therefore, the effect of changes in linoleic acid on UCP, after GIK administration, remains to be further studied. Linoleic acid is a precursor of arachidonic acid [27], which is known to induce uterine inflammation and contraction. However, this study found that the use of GIK, as opposed to oxytocin alone, reduced the production of 19R-hydroxy PGF_2α_ (a derivant of PGF_2α_ [28]) and effectively alleviated UCP. Furthermore, oleic acid can be converted into linolenic acid, which can then be converted into DHA substances. Compared to using only oxytocin, GIK could increase the production of DHA substances (16,17-EpDPE and 8-HDoHE). DHA has a beneficial effect on microcirculation and can reduce inflammatory responses [29, 30]. Therefore, the relief of UCP by GIK may be due to an increase in anti-inflammatory substances of DHA (16,17-EpDPE and 8-HDoHE) and a decrease in pro-inflammatory substances of 19R-hydroxy-PGF_2α_.

This study explore therapeutic strategies for UCP from the perspective of its pathogenesis and providing a new approach for its treatment. The GIK regimen, which encompasses glucose, potassium, and insulin, emerges as a cost-effective solution. Its deployment for myocardial safeguarding in clinical contexts over extensive periods substantiates its safety profile. For patients administered oxytocin to facilitate uterine involution in clinical settings, particularly those with intensified UCP such as individuals undergoing repeat delivery, the adjunct use of GIK alongside conventional analgesics may provide relief from UCP.

The study had some limitations. First, only energy metabolism and oxidized lipid metabolites were detected in this study; however, GIK may also act through other metabolic pathways. Second, uterine blood flow and oxygen levels were not monitored in this study because the animals were in an active state and could not be easily fixed for probing.

## Conclusion

This study confirmed the analgesic effect of GIK during postpartum oxytocin infusion. Metabolomics and glycolysis product analysis suggest that GIK’s alleviation of UCP is associated with its enhancement of glycolysis and its influence on phenylalanine synthesis, aspartate metabolism, and arginine synthesis pathways to improve uterine microcirculation or energy metabolism. Additionally, the effects of GIK appears to be linked to its influence on the linoleic acid metabolic pathway, leading to the increased production of DHA-derived anti-inflammatory substances 16,17-EpDPE and 8-HDoHE and a reduction in levels of pro-inflammatory substance 19R-hydroxy PGF_2α_.

## Electronic Supplementary Material

Below is the link to the electronic supplementary material.

Supplementary Material 1

Supplementary Material 2

## Data Availability

All data can be acquired from the corresponding author upon reasonable request.
