# Supplementary material for: Inhibitory Effect and Mechanism upon Glucose-Insulin-Potassium Administration on Postpartum Mice with Uterine Cramping Pain
Source: Reprod Sci. 2024 May 14;31(9):2741–52. doi: 10.1007/s43032-024-01579-8 (PMC11393204; doi:10.1007/s43032-024-01579-8)
Supplement: Supplementary file 1 — Supplementary Material 1 [file 43032_2024_1579_MOESM1_ESM.doc]

**Supplementary Content**

**eFig. 1** Overall process of the experiment.

**eFig. 2** TIC overlap diagram.

**eFig. 3** Violin diagram of different metabolites between group G and group C(ng/g, n=6).

**eFig. 4** Violin diagram of different metabolites between group O and group C(ng/g, n=6).

**eFig. 5** Violin diagram of different metabolites between group G and group O(ng/g, n=6).

**eFig. 6** TIC overlap diagram.

**eFig. 7** Violin diagram of different metabolites between groups G and C((nmol/g, n=6).

**eFig. 8** Violin diagram of different metabolites between groups O and C((nmol/g, n=6).

**eFig. 9** Violin diagram of different metabolites between groups G and O((nmol/g, n=6).

**eAppendix 1.** **Metabolomics targeting for metabolite detection and screening**

**eAppendix 2. Detection of glycolysis-related substances**

**eFig. 1** Overall process of the experiment.

**
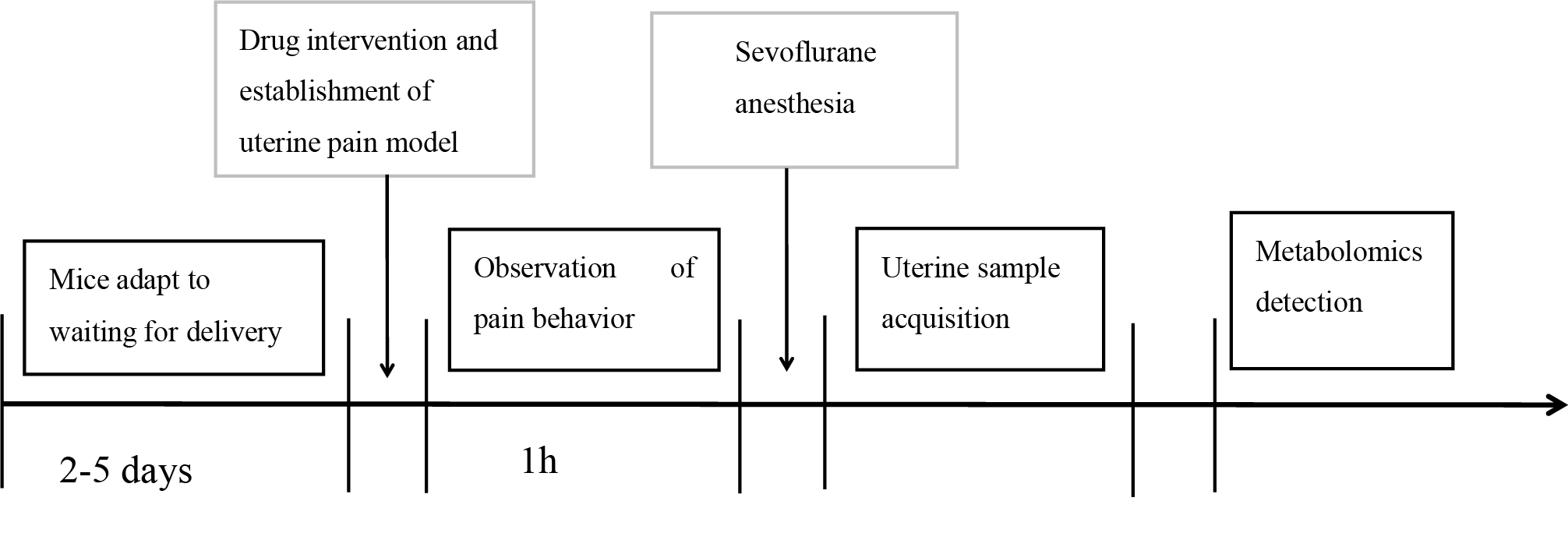
**

**eFig. 2** TIC overlap diagram.


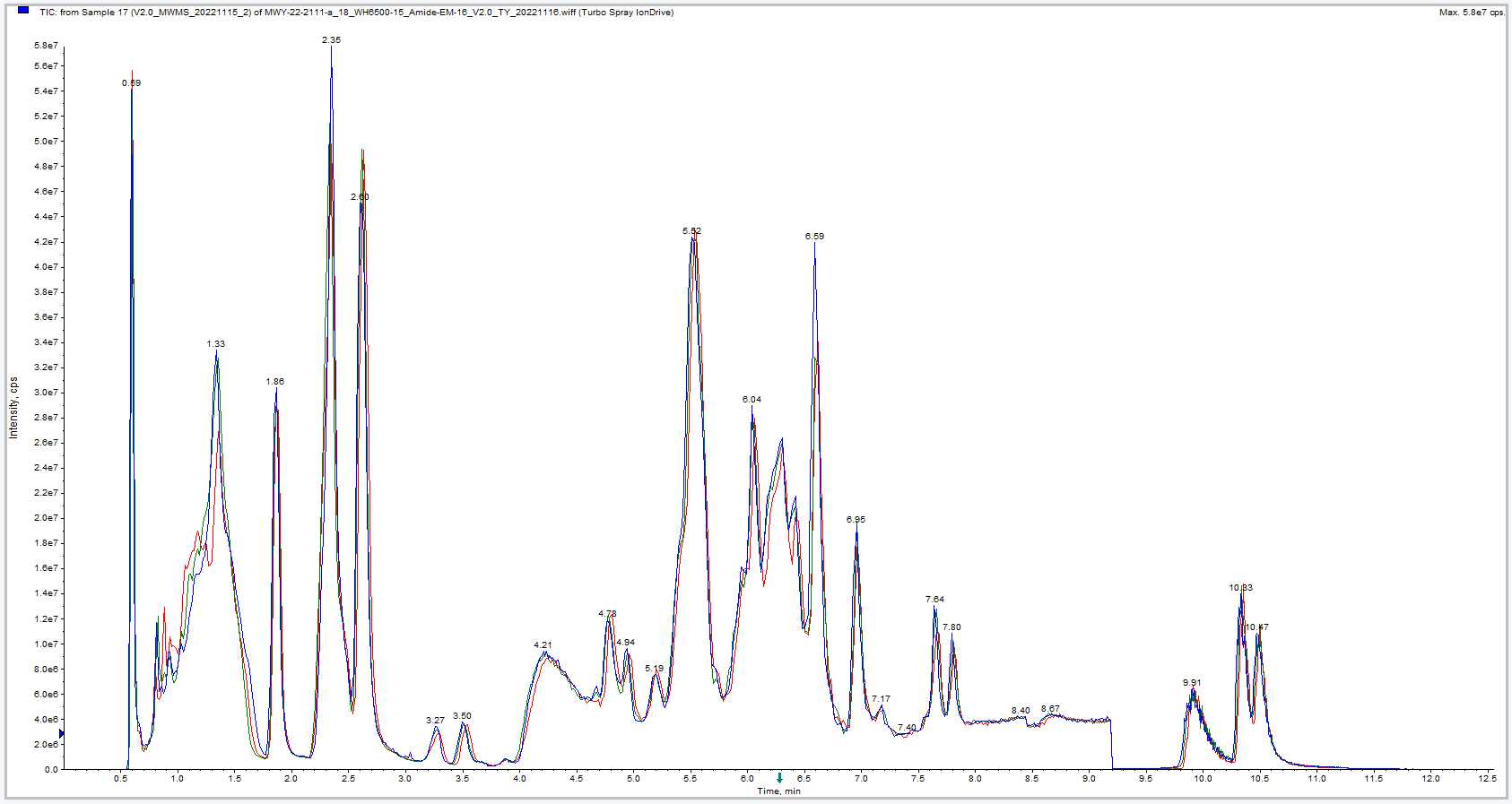


TIC, Total Ion Chromatogram.

**eFig.** **3** Violin diagram of different metabolites between groups G and C(ng/g, n=6).


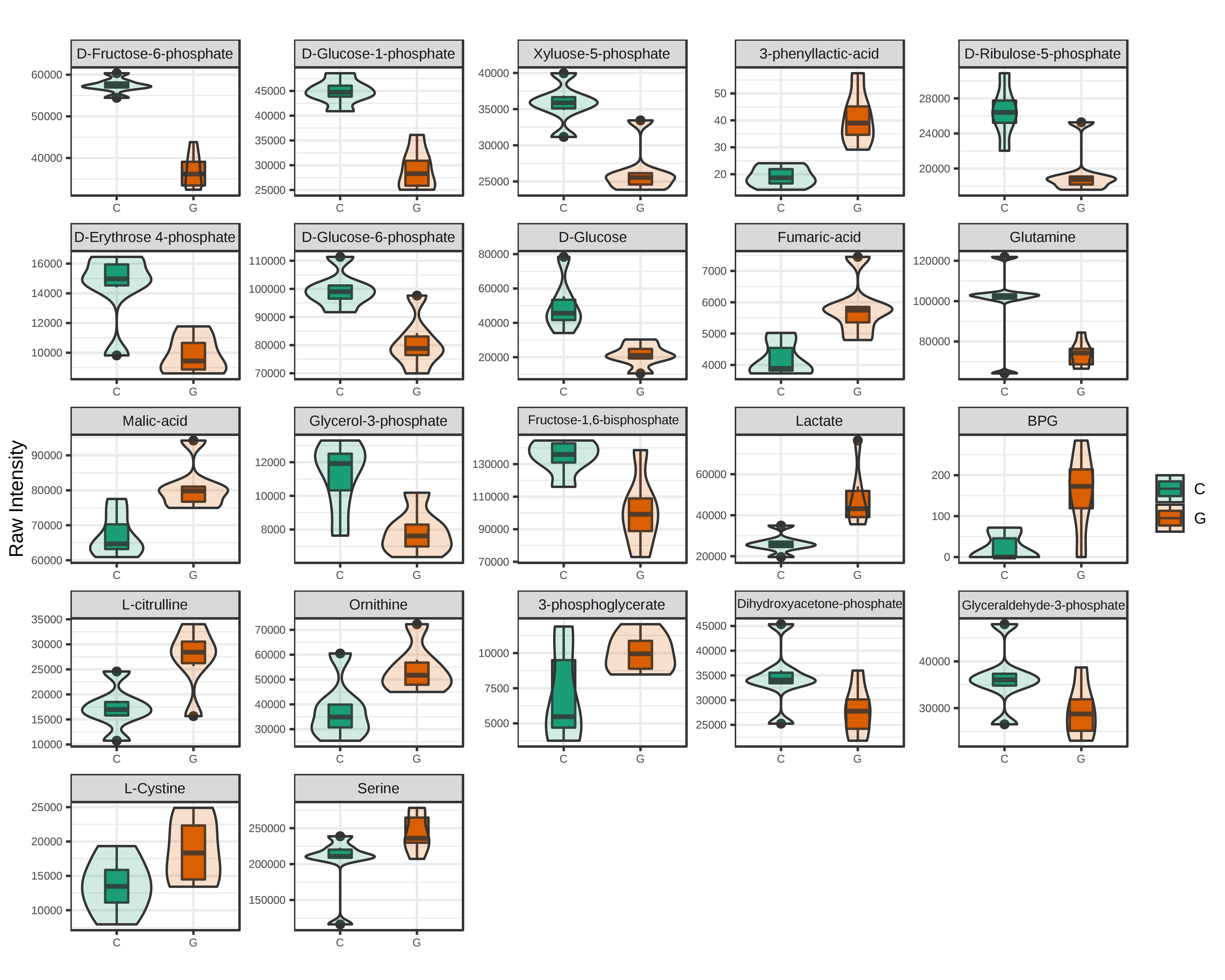


Screening criteria entailed a *P*-value < 0.1 and the | FC | ≥ 1.2.

**eFig. 4** Violin diagram of different metabolites between groups O and C(ng/g, n=6).


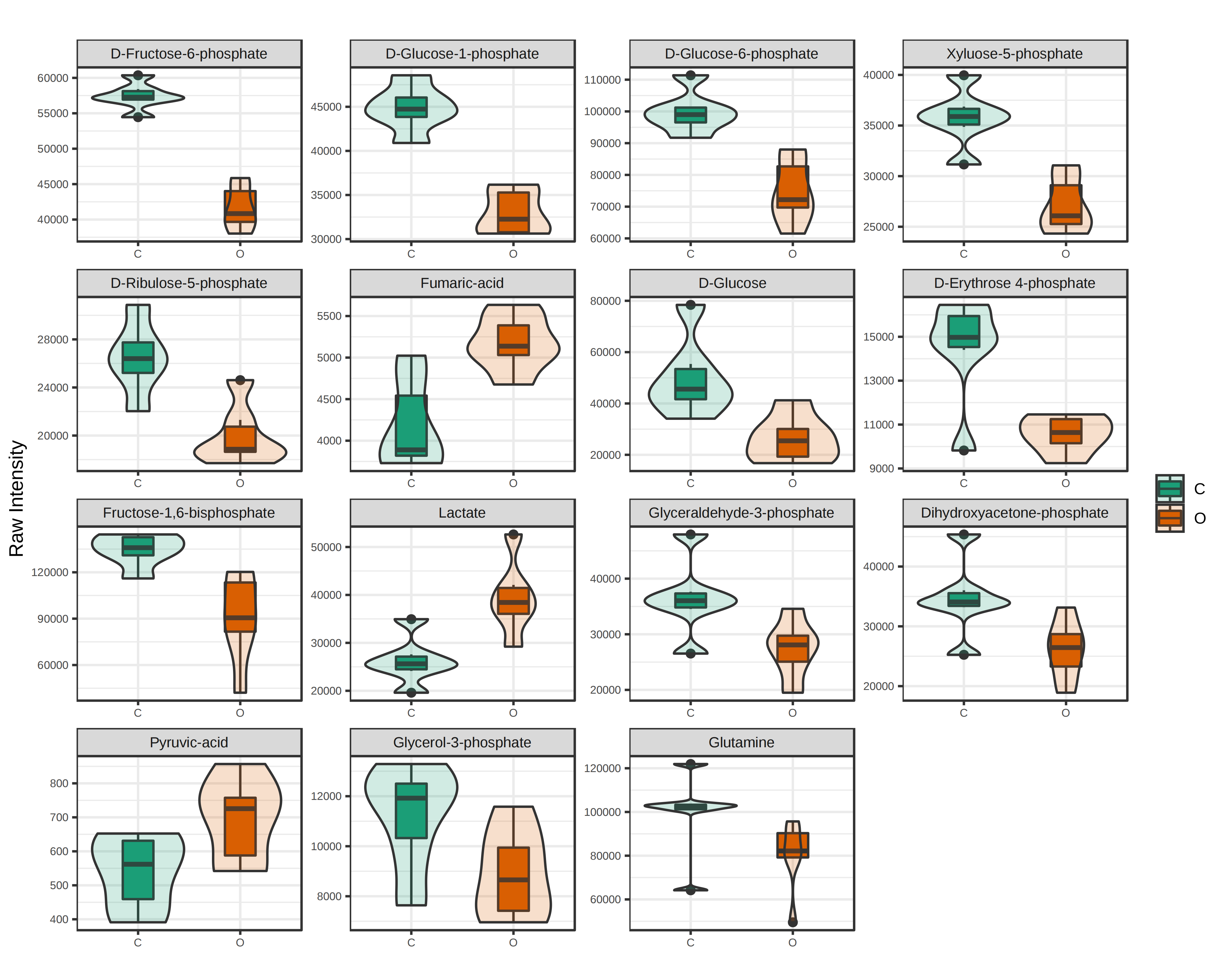


Screening criteria entailed a *P*-value < 0.1 and the | FC | ≥ 1.2.

**eFig. 5** Violin diagram of different metabolites between groups G and O(ng/g, n=6).


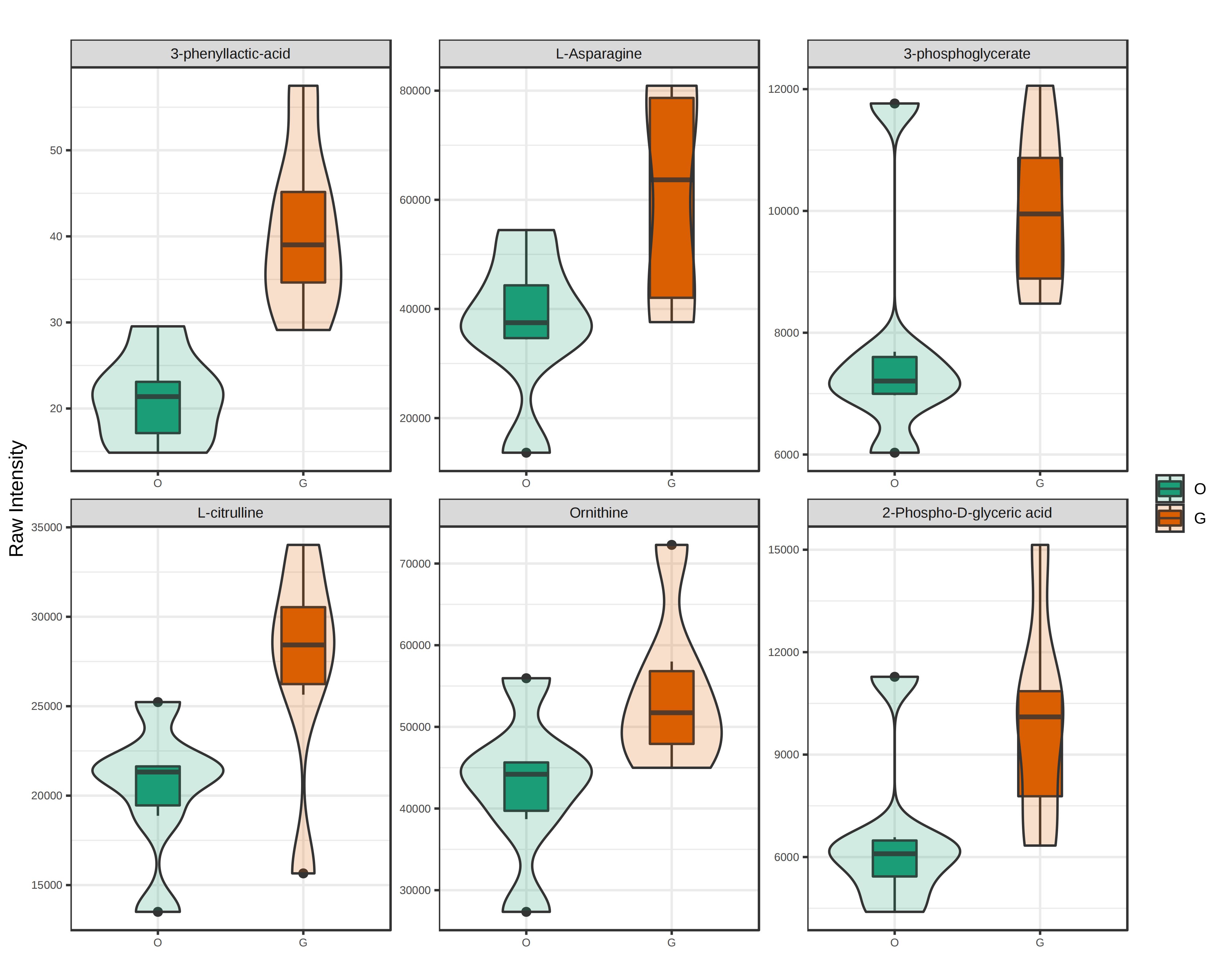


Screening criteria entailed a *P*-value < 0.1 and the | FC | ≥ 1.2.

**eFig. 6** TIC overlap diagram.


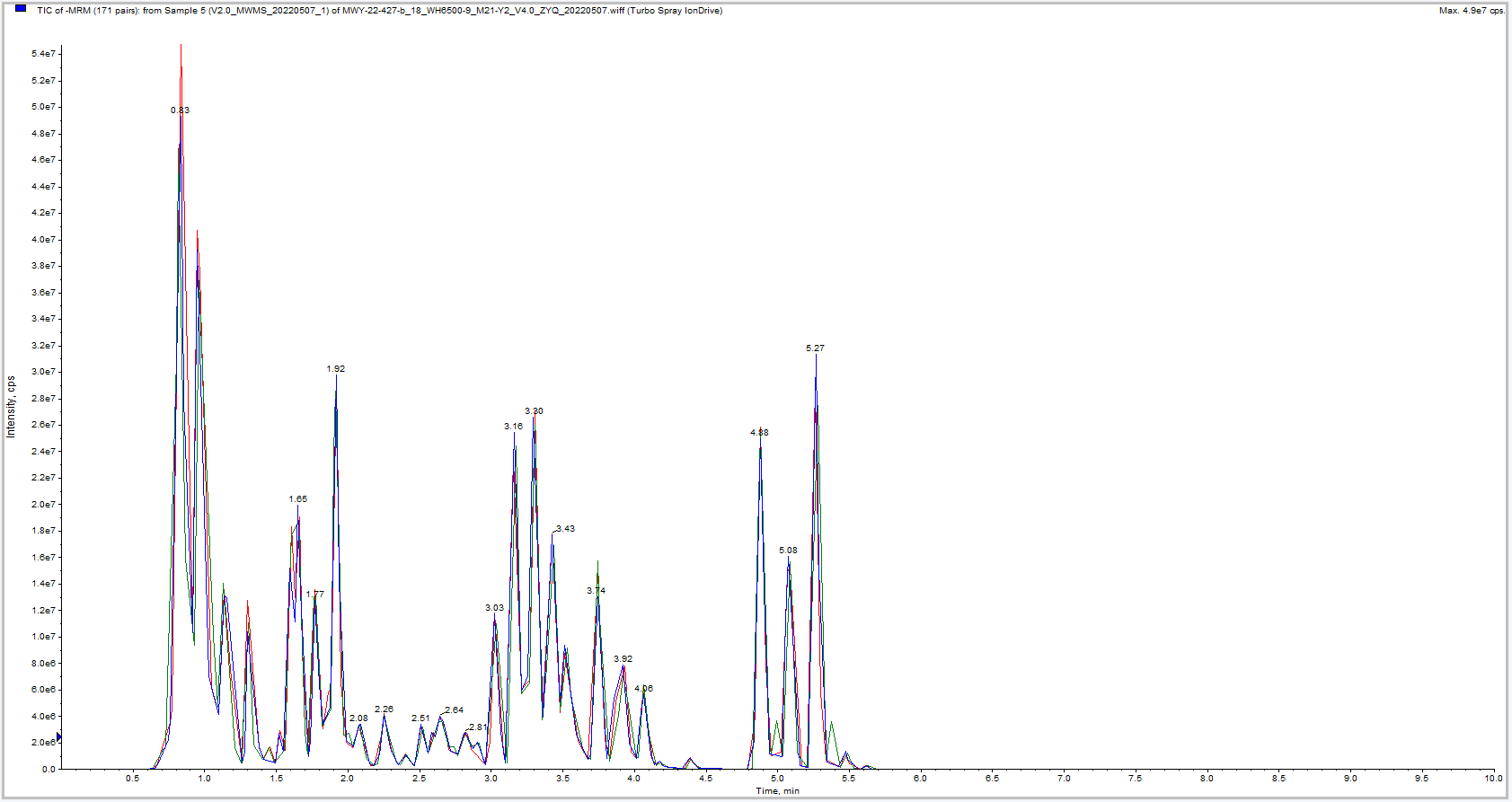


TIC, Total Ion Chromatogram.

**eFig. 7** Violin diagram of different metabolites between groups G and C((nmol/g, n=6).
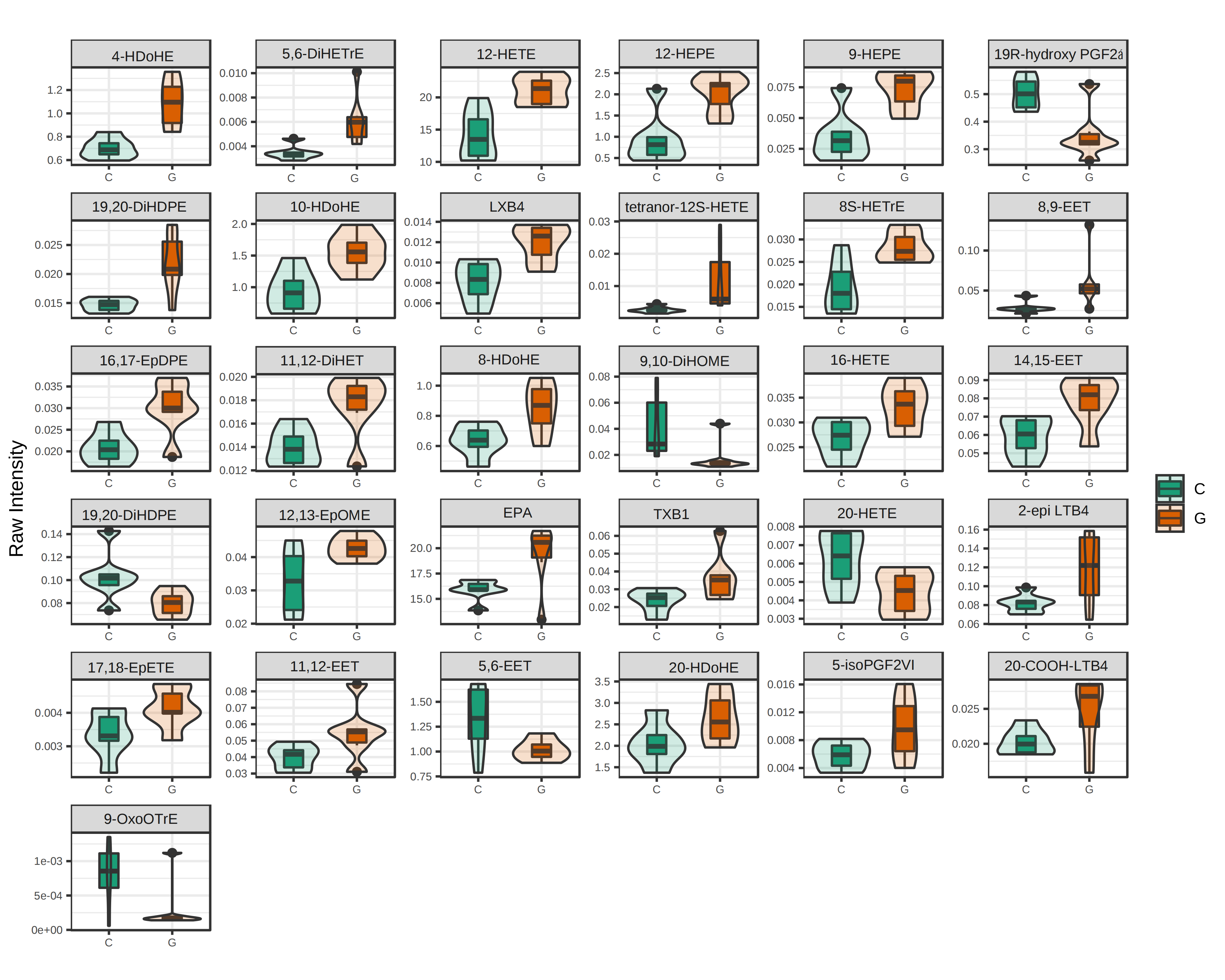


Screening criteria entailed a *P*-value < 0.1 and the | FC | ≥ 1.2.

**eFig. 8** Violin diagram of different metabolites between groups O and C((nmol/g, n=6).
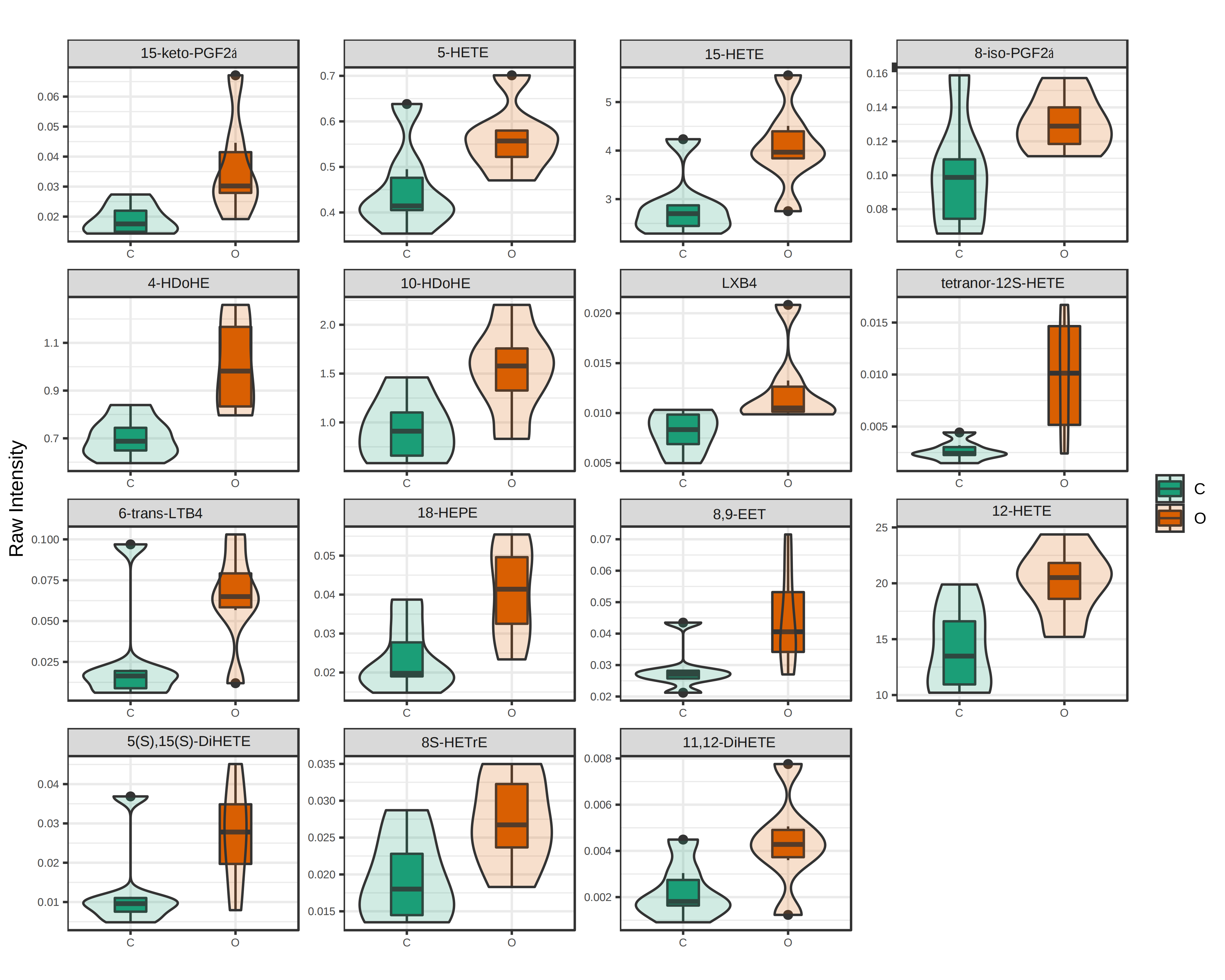


Screening criteria entailed a *P*-value < 0.1 and the | FC | ≥ 1.2.

**eFig. 9** Violin diagram of different metabolites between groups G and O((nmol/g, n=6).
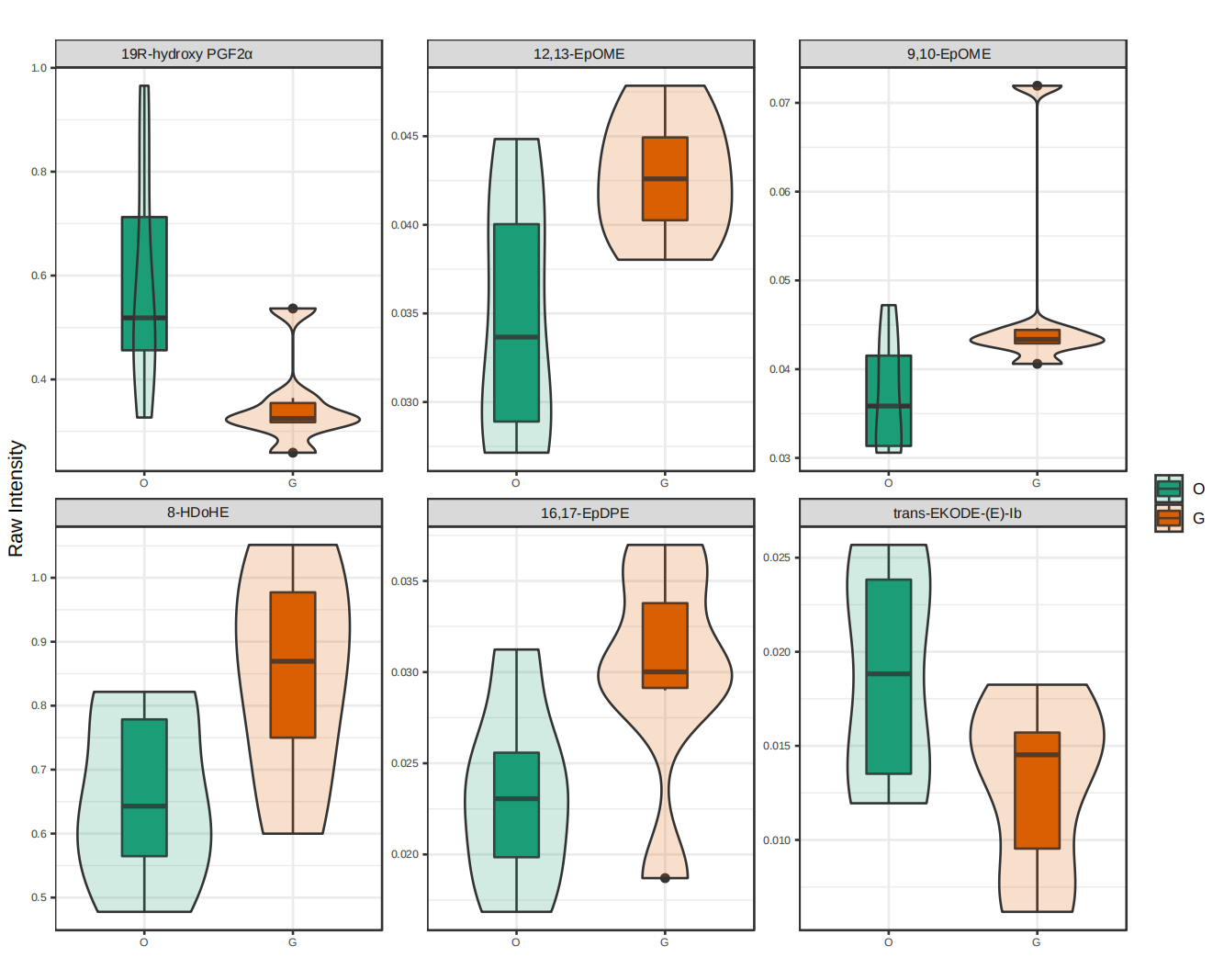


Screening criteria entailed a *P*-value < 0.1 and the | FC | ≥ 1.2.

**eAppendix 1. Metabolomics targeting for metabolite detection and screening**

**1.Energy** **metabolite detection**

**1.1Extraction of the target compound**

Fifty milligrams of the samples were weighed and added into a 2 mL centrifuge tube. The weight of each sample was recorded. Subsequently, the weighed samples were each mixed with 500 μL of 70% methanol-water extract that was pre-cooled at –20 ℃. The mixture was swirled for 3 minutes and centrifuged for 10 minutes at 4 ℃ and 12000 rpm. The supernatant was carefully collected and transferred to a 1.5 mL centrifuge tube. The tube was left in a –20 ℃ refrigerator for 30 minutes and then centrifuged again for 10 minutes at 12000 rpm and 4 ℃. Finally, 200 μL of the supernatant was taken from the protein precipitation plate for analysis using the machine (SCIEX Corporation, USA), and stored in a refrigerator at –20 ℃.

**1.2 Chromatography-mass spectrometry acquisition conditions**

The primary data acquisition instrument systems utilized in this study were those for Ultra Performance Liquid Chromatography (UPLC) and Tandem Mass Spectrometry (MS/MS). The UPLC system employed a Waters ACQUITY UPLC BEH Amide column with a particle size of 1.7 µm and dimensions of 100 mm×2.1 mm i.d. The mobile phases comprised ultra-pure water, which contained 10 mM ammonium acetate and 0.3% ammonia water (phase A), and 90% acetonitrile/water in a volume-to-volume ratio (phase B). The flow rate was set at 0.40 mL/min, and the column temperature was maintained at 40 ℃. A sample volume of 2 µL was injected into the system. For the mass spectrometry component, the electrospray ionization (ESI) source temperature was set at 550 ℃, and the mass ionization voltage was configured at 5500 V in positive ion mode and –4500 V in negative ion mode. The Curtain Gas (CUR) was set at 35 psi. Each ion pair in Q-Trap 6500+ was scanned and detected based on their optimized Declustering Potential (DP) and Collision Energy (CE).

**1.3 Sample quality control**

By overlapping display of the Total Ion Chromatogram (TIC) from the same quality control sample in mass spectrometry analysis, the results of our analysis showed that the retention time and peak intensity of detected metabolites in the TIC were consistent. The high curve overlap indicates good system stability during the detection of the same sample at different times using mass spectrometry, as illustrated in eFig.2 in Supplement.

**2 Oxidized lipid metabolite detection**

**2.1 Extraction of the target compound**

Twenty milligrams of the samples were mixed with internal standard extract, 80 μL ultra-pure water, and 200 μL internal standard extract (methanol/acetonitrile [1:1, V/V]), before being homogenized by a ball mill. The mixture was then swirled for 5 minutes and stored in a –20 ℃ refrigerator for 30 minutes to precipitate the proteins. After centrifugation at 4℃ and 12000 rpm for 10 minutes, the supernatant was collected and extracted again. The two supernatants were combined and collected using a solid-phase extraction column through the steps of leaching, washing, and stripping. The eluent was concentrated, dried, and subsequently re-dissolved in 100 μL methanol/water. The solution was then swirled for 30 seconds to produce the supernatant for use in the chromatographic mass spectrometry analysis.

**2.2Chromatography-mass spectrometry acquisition conditions**

The data acquisition instrument systems used in this study included those for UPLC and MS/MS. The UPLC system employed a Waters ACQUITY UPLC HSS T3 C18 column measuring 1.8 µm, 100 mm × 2.1 mm i.d. The system utilized two mobile phases, acetonitrile/water (60/40, V/V) for phase A and acetonitrile/isopropyl alcohol (50/50, V/V) for phase B. The flow rate was 0.4 mL/min, and the UPLC system had a column temperature of 40 ℃. Moving on to the mass spectrometry component, the ESI source temperature was set at 550 ℃, and the mass spectrometry voltage was -4500 V. The CUR in the system was maintained at 35 psi. Each ion pair in Q-Trap 6500+ was scanned and detected based on its optimized DP and CE values.

**2.3 Sample quality control**

The results of the TIC analysis indicated that the retention time and peak intensity of the metabolites, detected in the total ion current, were consistent. Additionally, the high degree of curve overlap suggested excellent system stability when the mass spectrum detected the same sample at different times, as illustrated in eFig.6 in Supplement.

**3** [**Data screening**](javascript:;)

This study utilized a combination of multivariate statistical analysis and univariate statistical analysis. Multivariate statistical analysis employed principal component analysis (PCA), an unsupervised pattern recognition method, which was observed using R software (www.R-Project.org/). Univariate statistical analysis involved hypothesis testing and fold change (FC) analysis. | FC | and *P*-values were utilized to screen for differentially metabolized products between different groups.

Screening criteria: For comparing each pair of groups, metabolites with *P* < 0.1 were selected; on the basis of the former selection, metabolites with FC ≥ 1.2 and FC ≤ 0.83 were also selected.

**Appendix2 Detection of glycolysis-related substances**

**1 Chemiluminescence to measure ATP levels**

Uterine tissues were lysed, and the supernatant was collected. ATP standard solution was added to the lysis solution for constructing a standard curve. The ATP detection reagent and standards were added to the wells, and chemiluminescence was measured using a multifunctional microplate reader (Bioteck Corporation, USA). ATP concentrations in the samples were calculated based on the standard curve.

**2 Spectrophotometry to determine lactate concentration**

Extraction was performed, and after centrifugation of homogenized samples, the supernatant was collected. Standard solutions were prepared, and a standard curve was plotted. Subsequently, the samples, standard solutions, and reagents were added and centrifuged. The absorbance was measured at 570 nm, and lactate content in the samples was calculated based on the standard curve.

**3 Spectrophotometric determination of LDH relative content**

After lysing uterine tissues and obtaining the supernatant, LDH detection working solution was prepared according to the instructions(Shanghai Biyuntian Biotechnology Co., LTD., China). The LDH detection working solution was added to the respective wells, mixed, and incubated. Absorbance was measured at 490 nm with a reference wavelength of 600 nm for dual-wavelength measurement. The relative LDH content was calculated with group C as the standard, and other groups were compared to group C.
