# Supplementary material for: Inhibitory Effect and Mechanism upon Glucose-Insulin-Potassium Administration on Postpartum Mice with Uterine Cramping Pain
Source: Reprod Sci. 2024 May 14;31(9):2741–52. doi: 10.1007/s43032-024-01579-8 (PMC11393204; doi:10.1007/s43032-024-01579-8)
Supplement: Supplementary file 2 — Supplementary Material 2 [file 43032_2024_1579_MOESM2_ESM.doc]

Apr. 4, 2024

Warren Ben Nothnick

Associate Editor

Reproductive Sciences

Dear Editor:

I wish to re-submit the manuscript titled “**Inhibitory effect and mechanism upon glucose-insulin-potassium administration on postpartum mice with uterine cramping pain**.” The manuscript ID is *Reproductive Sciences*.

We thank you and the reviewers for your thoughtful suggestions and insights. The manuscript has benefited from these insightful suggestions. I look forward to working with you and the reviewers to move this manuscript closer to publication in the  *Reproductive Sciences.*

The manuscript has been rechecked and the necessary changes have been made in accordance with the reviewers’ suggestions. The responses to all comments have been prepared and attached herewith.

Thank you for your consideration. I look forward to hearing from you.

Sincerely,

Hong Li

Department of Anesthesiology, Second Affiliated Hospital of Army Medical University, University, No. 183, Xinqiao Street, Shapingba, Chongqing, 400037, China

Tel: +86-13608380123

Fax: +86-02368774997

[lh78553@163.com](mailto:lh78553@163.com)
